# Leflunomide-Induced Weight Loss: Involvement of DAHPS Activity and Synthesis of Aromatic Amino Acids

**DOI:** 10.3390/metabo14110645

**Published:** 2024-11-20

**Authors:** Xiaoyu Guo, Kai Wang, Hongli Chen, Na Wang, Dongmei Qiu, Haiyun Huang, Jiyu Luo, Ao Xu, Lingyun Xu, Zejun Yu, Yuanyuan Li, Hongling Zhang

**Affiliations:** 1 School of Life Science and Technology, Wuhan Polytechnic University, Wuhan 430023, China; xiaoyuguo2000@outlook.com (X.G.); wangkai@hnse.org (K.W.); chl3150766568@outlook.com (H.C.); w3036576087@outlook.com (N.W.); doctorxly9898@163.com (L.X.); yuzejun@126.com (Z.Y.); 2 School of Medicine and Health, Wuhan Polytechnic University, Wuhan 430023, China; qdm520qiu@outlook.com (D.Q.); amyhuang9912@gmail.com (H.H.); jiyuluo0615@outlook.com (J.L.); aoxu0624@outlook.com (A.X.); 3 School of Public Health, Tongji Medical College, Huazhong University of Science and Technology, Wuhan 430030, China; 4 Key Laboratory of Environment and Health, Ministry of Education & Ministry of Environmental Protection, and State Key Laboratory of Environmental Health (Incubation), School of Public Health, Tongji Medical College, Huazhong University of Science and Technology, Wuhan 430030, China

**Keywords:** leflunomide, kidney injury, aromatic amino acids, weight loss

## Abstract

**Background/Objectives:** Leflunomide, an isoxazole immunosuppressant, is widely used in the treatment of diseases such as rheumatoid arthritis (RA) and psoriatic arthritis (PsA) as well as lupus nephritis (LN). In recent years, clinical data have shown that some patients have obvious weight loss, liver injury, and other serious adverse reactions after taking leflunomide. However, the causes and mechanisms by which leflunomide reduces weight are unclear. **Methods:** Therefore, we used a mouse animal model to administer leflunomide, and we observed that the weight of mice in the leflunomide experimental group was significantly reduced (*p* < 0.01). In this animal experiment, a metabolomic method was used to analyze the livers of the mice in the experimental group and found that the main difference in terms of metabolic pathways was in the metabolism of aromatic amino acids, and it was confirmed that leflunomide can inhibit the limitations of phenylalanine, tyrosine, and tryptophan biosynthesis. **Results:** Our study revealed that leflunomide inhibited the activity of DAHPS in the gut microbiota, disrupting the metabolism of phenylalanine, tyrosine, and tryptophan, as well as the metabolism of carbohydrates and lipids. Leflunomide also increased endoplasmic reticulum stress by activating the PERK pathway, thereby promoting CHOP expression and increasing apoptosis-induced liver damage. **Conclusions:** These effects may be related to the observed weight loss induced by leflunomide.

## 1. Introduction

Leflunomide is an isoxazole immunosuppressive agent, and its chemical structure is an isoxazole derivative (N-[4-trifluoromethylphenyl]-methylisoxazole-4-carboxamide). Leflunomide has been widely used in the clinical treatment of rheumatoid arthritis (RA), showing definite efficacy in psoriatic arthritis, lupus nephritis [1], vasculitis [2], and transplantation immunity, and has shown antitumor and antiviral efficacy [3]. Adverse reactions that have been reported include gastrointestinal upset, liver injury, weight loss, hypertension, headache, hepatotoxicity, and hair loss, as well as a predisposition to infection and peripheral neuropathy [4].

In clinical settings, weight loss has been observed with leflunomide. For instance, a female who took leflunomide for psoriatic arthritis experienced abdominal pain and significant weight loss after 18 months, and the symptoms improved after 20 days of discontinuation [5]. Esfahani et al. reported severe diarrhea and weight loss in a male with multiple sclerosis six months after initiating teriflunomide use [6]; teriflunomide is an active metabolite of leflunomide. Coblyn and colleagues followed 70 patients with rheumatoid arthritis who had been taking leflunomide for more than 1 year and found that 7.1% of them had significant weight loss compared with the pre-drug period [7]. Coblyn and colleagues hypothesized that the possible mechanism for this side effect is that the inhibition of Dihydroorotate dehydrogenase (DHODH) activity by leflunomide interferes with pyrimidine synthesis in lymphocytes while exerting a non-specific inhibitory effect on normal energy metabolism in the mitochondria of the rest of the body, thereby causing weight loss. However, these assumptions are not supported by experimental data, and the mechanism by which leflunomide causes weight loss is unclear. This study will discuss the mechanisms by which leflunomide causes weight loss in patients. A previous article clearly states that a patient experienced weight loss after taking leflunomide and that it was not the combination of other medications that caused this adverse reaction [8]. The main purpose of this study is to find the possible mechanism of leflunomide-induced weight loss to provide safety for the clinical use of the drug at a later stage.

Leflunomide undergoes nearly complete hydrolysis to a single active metabolite, known as A771726 or teriflunomide, during first-pass metabolism in the intestines and liver [9]. DHODH is an essential enzyme in the biosynthesis of pyrimidine nucleotides and is a known therapeutic target for various diseases. Both leflunomide and teriflunomide are effective inhibitors of DHODH [10], inhibiting lymphocyte proliferation [11]. Leflunomide is completely metabolized to A771726 in the liver and intestines after oral administration, with peak blood concentrations reached after 6 h of oral intake. A771726 undergoes further metabolism in the body, being cleared by bile and kidneys. Adverse reactions associated with leflunomide in clinical trials include diarrhea [12], but so far, there is no evidence linking the risk of weight loss with diarrhea caused by leflunomide.

Various autoimmune diseases may occur when the gut microbiota becomes imbalanced. Wang and colleagues [13] provided evidence that short-chain fatty acids are highly correlated with RA at the genetic, functional, and phenotypic levels, indicating a strong gut microbiota–immune joint interaction in RA patients. The human gut microbiota can influence host physiological functions by regulating various processes such as inflammation, oxidative stress, nutrient absorption [14], and immune modulation [15]. Nutrient absorption is fundamental to maintaining health, and the gut microbiota facilitates the breakdown and metabolism of nutrients for absorption, such as the essential amino acid tryptophan [16] and short-chain fatty acids [17]. The essential amino acids phenylalanine, tyrosine, and tryptophan are aromatic amino acids, and their absorption relies on the assistance of the gut microbiota [18,19]. 3-Deoxy-D-Arabino-Heptulosonate 7-Phosphate Synthase (DAHPS) is the first enzyme of the shikimate pathway and one of the rate-limiting enzymes in the synthesis of aromatic amino acids [20]. In Escherichia coli, three genes encoding DAHPS were inhibited by inhibitors of tyrosine and phenylalanine. In plants, redox regulation can modulate the activity of the DAHPS enzyme; compounds such as DTT and mercaptoethanol were found to activate DAHPS [21].

In our study, the mechanisms of weight loss in patients following leflunomide administration are investigated. Additionally, metabolomic analysis is employed to identify differential metabolites in mice treated with leflunomide. These metabolites are mapped to specific KEGG metabolic pathways, and enrichment analysis is conducted to identify differential pathways, elucidating the reasons for weight loss in the experimental mice.

## 2. Materials and Methods

### 2.1. Animals and Cells

This study was approved by the Animal Experimental Ethics Committee of Wuhan Polytechnic University and was conducted in accordance with the guidelines of Care and Use of Laboratory Animals published by the China National Institute of Health.

SPF Kunming mice weighing 18–21 g (9–10 weeks old) were purchased from the Hubei Center for Disease Control and Prevention; half were male and half were female, and they were bred at the Experimental Animal Center of Huazhong University of Science and Technology under specific pathogen-free conditions. We gave the mice feed and drinking water, the mice were free to eat and drink, and the padding was changed every 3 days. They were kept in an animal room with alternating light and dark cycles, changing every twelve hours, day and night, and the room temperature was 22 ± 2 °C.

Normal human immortalized hepatocytes came from Wuhan University (Wuhan, China). Human immortalized hepatocytes were cultured in a low-glucose DMEM (Dulbecco’s modified Eagle medium) containing 10% fetal bovine serum, 100 U/mL penicillin, and 100 U/mL streptomycin in a 37 °C, 5% CO_2_ incubator. Human normal hepatocytes were seeded in 96-well cell culture dishes or 60 mm cell culture dishes at 3–5 × 105 cells/mL density.

After 15 days of administration (or after the body weight of the mice in the administration group was observed to decrease significantly), the eyeballs were removed and the blood was disposed of to avoid hemolysis. The serum was separated and frozen at −80 °C after packing. The mice were dissected using an autoclaved surgical instrument, and the lungs, livers, and kidneys of the mice were taken, weighed, and cryopreserved at −80 °C. The liver was divided into two parts: one part was frozen, and the other part was fixed with 10% formaldehyde for 48 h to make paraffin sections. After the experiment concluded, the mice were euthanized by cervical dislocation. Epididymal, renal, and intestinal fat were collected from male mice, while intestinal systems and perirenal fat were collected from female mice. The samples were weighed and recorded, and the total fat and body fat percentages were calculated using the following formula: Male total fat = epididymis, intestinal fat + perirenal fat, female total fat = abdominal fat including ovarian fat + perirenal fat, fat body ratio = total fat/body weight. Before we performed this calculation, we ensured that the fat was dried with absorbent paper to absorb water and blood. 

### 2.2. Reagent

Leflunomide (98%, Tokyo Chemical Co., Ltd., Tokyo, Japan); sodium carboxymethyl cellulose (CMC, viscosity 300–800, Shanghai Yuanye Chemical Co., Ltd., Shanghai, China); and dimethyl sulfoxide (DMSO) from Sigma-Aldrich Corporation (obtained from St. Louis, MO, USA) were used. Fetal bovine serum (FBS) was purchased from Atlanta Biological Products (Lawrenceville, GA, USA); antibiotic–antifungal drugs were obtained from Biotech (Grand Island, NY, USA). PERK (1:1000), CHOP (1:1000), phospho-eIF2α (97211; 1:1000), caspase-3 (1:1000), and GAPDH (sc-47724; 1:2000), came from Santa Cruz. A CCK-8 (Merck KGaA, Beijing, China) kit was used. The mouse DAHPS vitality test kit came from Shanghai Jianglai Biological Company.

### 2.3. Animal Grouping

Mice were randomly divided into four groups (n = 16): a negative control group and three leflunomide experimental groups. Experimental groups included groups treated with low-, medium-, and high-dose leflunomide (3 mg/kg; 10 mg/kg; 30 mg/kg). To avoid the influence of different genders on drug metabolism, we divided each group of mice into 8 females and 8 males. The negative control group was given the corresponding volume of CMC solution every day. For the preparation of the CMC solution, 1 g of CMC powder was added to 100 mL of warm (40–50 °C) pure water and stirred until dissolved. The leflunomide experimental group was given different drug doses (30 mg/kg/d; 10 mg/kg/d; 3 mg/kg/d). For the preparation of the leflunomide suspension, leflunomide powder was mixed with 1% CMC solution by ultrasonic mixing. Due to the limited gastric volume, when the volume was not more than 30 g, the concentration of the leflunomide solution was 2 mg/mL; when the body weight exceeded 30 g, a 4 mg/mL solution was prepared for gavage. The mice were weighed at the same time every morning before administration. The mice’s body weight and food intake were continuously recorded for 12 days.

### 2.4. Immunohistochemical Method

At the end of the experiment, 4 liver tissues of mice in the control group and 4 liver tissues of mice in the administration group were taken. A total of 16 liver tissues were used to detect liver damage, polysaccharide content, and fat content in the liver. The tissue was fixed with formaldehyde and sealed with wax blocks. After dewaxing, the tissue was stained with hematoxylin and eosin (HE). Then, the polysaccharides in the tissues were stained by periodate Schiff staining (PAS) to observe the changes in polysaccharides in the tissues. Triglycerides in liver tissue were stained with oil red (OR) to observe tissue fat content changes. The protein was stained with Ki67 and Calpain I to observe the protein content and distribution. Then, the sections were photographed under an inverted microscope. The degree of staining was analyzed by ImageJ (1.8.0).

### 2.5. Metabolomic Analysis of Liver Flux Target

Liver samples from mice were divided into two groups, a control group and a treatment group, with 10 biological replicates in each group, totaling 20 samples. High-throughput targeted metabolomic analysis was conducted. Quality control (QC) samples were tested at the beginning and end of each day, and 1 QC sample was inserted after every 10 samples. Additionally, a blank sample was inserted after every 10 samples to prevent carryover effects. The raw data included 4 QC samples and 20 experimental samples, from which 168 peaks were extracted. To enhance data analysis, a series of preprocessing steps were performed on the raw data, including peak filtering, missing value imputation, and data normalization. After preprocessing, 168 peaks were retained. The processed data were then subjected to principal component analysis (PCA) and orthogonal partial least squares-discriminant analysis (OPLS-DA) using SIMCA software (V14.1, Sartorius Stedim Data Analytics AB, Umea, Sweden). Differential metabolites between the two groups were identified and mapped to their respective biochemical pathways through metabolic enrichment and pathway analysis based on database searches (KEGG, http://www.kegg.jp/kegg/pathway.html, accessed on 17 November 2019).

### 2.6. Cell Function Test

Cytotoxicity assay: Normal human hepatocytes were plated in 96-well plates at a concentration of 1 million/mL per well and 100 μL per well and divided into the untreated group (UT), DMSO (20 μM), and leflunomide treatment group—20 μM, 50 μM, 100 μM—and cultured for 12 h. CCK-8 (Sigma) kit measurement takes 100 µL of supernatant and measures the OD value at 450 nm absorbance; the results are expressed as mean OD value ± SD.

Cell proliferation assay: Normal human hepatocytes were spread on 60 mm plates, with 50,000 per well. The control group was treated with DMSO, and the treatment group was treated with 20 μM, 50 μM, and 100 μM leflunomide solution (leflunomide dissolved in DMSO), with 1.5 mL of medium per well. Cell counts were performed on days 3, 6, and 9. Cells were cultured with the drug-containing medium every three days. We observed the changes in the number of cells in different concentration groups and control groups and collected data.

Cell cloning experiment: A total of 1000 normal human hepatocytes per well were placed in a six-well plate. After the cells adhered to the wall, the drug and control groups were treated. The medium was changed every three days, and the drug was added to each medium. After 9–12 days, the cell plate was taken out. First, 4% paraformaldehyde was placed in the refrigerator at −20 °C and fixed for 10 min, then rinsed with water. Then, 5% crystal violet was placed in a shaker at room temperature for 15 min. Then, the crystal violet was stained and then rinsed in water, after was placed on the cover or absorbent paper for drying. Pictures were taken after the water was dried.

### 2.7. Western Blot Analysis

Cell proteins were extracted using 2 × SDS lysis buffer (50 mM Tris-HCl pH 8.0, 1 mM EDTA, 250 mM NaCl, 1% NP-40, and 0.5% deoxycholate) in the presence of protease and phosphatase inhibitors (Thermo Fisher, Waltham, MA, USA). Total protein concentration was measured using the BCA Assay Kit (Thermo Fisher, USA) according to the manufacturer’s instructions. Fifteen micrograms of cell protein were separated by electrophoresis on a 10% SDS–polyacrylamide gel and transferred to a PVDF membrane. The membrane was blocked with 5% non-fat milk in Tris-buffered saline (TBS) containing 0.1% Tween-20 for 1 h and incubated overnight at 4 °C with primary antibodies: PERK (1:1000), CHOP (1:1000), phospho-eIF2α (97211; 1:1000), GAPDH (sc-47724; 1:2000), and caspase-3 (1:1000). After multiple washes, the membrane was incubated at room temperature for 1 h with horseradish peroxidase-conjugated secondary antibodies (anti-rabbit IgG or anti-mouse IgG, Sigma-Aldrich) in 5% non-fat milk. Immunoreactive bands were detected using the ECL Western blotting detection system according to the manufacturer’s instructions.

### 2.8. Isolation of Intestinal Flora Samples

The contents of small intestinal tissue and feces of mice were 0.2 g, respectively, and 4 samples were taken from the experimental group and the control group, and a total of 32 samples were obtained. First, 1 mL PBS was added to the centrifuge tube containing the fecal sample, fully mixed, and placed in a shaker at 37 °C for 10 min (speed: 180 rpm). After the warm bath, the supernatant was centrifuged at 12000 rmp for 1 min, the supernatant was discarded, and the lysate (20 mg/mL lysozyme, 0.5 mM EDTA, 10% Triton-X, 20 mM Tris-Hcl, PH adjusted to 8.0) was added. Finally, the supernatant was centrifuged at a constant speed of 3000 rpm for 10 min at 37 °C, and 50 uL of the supernatant was obtained as a sample for DAHPS activity determination.

### 2.9. Detection of DAHPS Activity

The activity of DAHPS in mice was measured using an activity assay kit (Jianglai Bio). Polyacrylamide microplates were coated with polyclonal antibodies against mouse DAHPS. Samples were added, and the optical density (OD) at 450 nm was measured using a microplate reader. According to the kit instructions, standard concentrations were set at 0 U/mL, 50 U/mL, 100 U/mL, 200 U/mL, 400 U/mL, and 800 U/mL. DAHPS activity in the samples was calculated using a standard curve plotted with these six concentrations.

### 2.10. Statistics

Statistical analyses were performed with SPSS 26. Each group of experimental data contained 6 repeated results, and the data were expressed as mean ± SEM. A one-way or two-way analysis of variance was used to compare the groups, followed by a Bonferroni post-hoc analysis. *p* < 0.05 was considered statistically significant.

## 3. Results

### 3.1. Effect of Leflunomide on Body Weight of Mice

**Figure 1:**
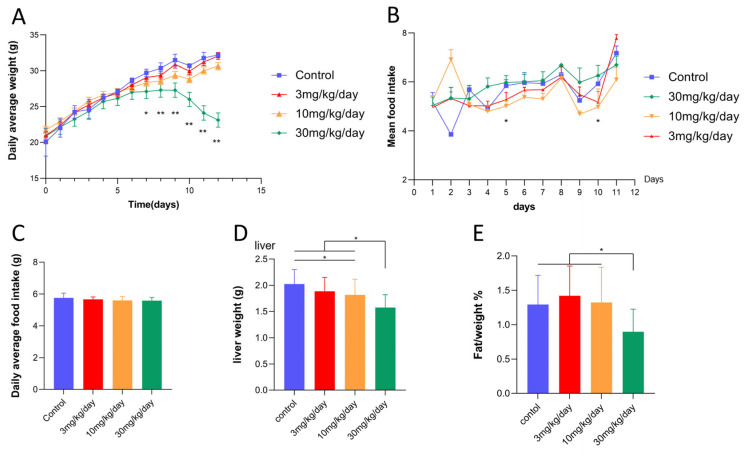
The body weight of mice in the leflunomide treatment group decreased significantly. (**A**) The average daily body weight change curve of the four groups of mice. (**B**) The average daily food intake of the four groups of mice. (**C**) Average 11-day food intake of each group (16 mice) of the four groups. (**D**) Liver weight changes of mice in the four groups after 12 days of drug treatment. (**E**) Four groups of mice were weighed and sacrificed 12 days after administration to obtain fat for calculating body fat ratio data. *, *p* < 0.05 and **, *p* < 0.01.

Following a seven-day leflunomide intervention, the body weight of mice in the high-dose group was significantly reduced compared to the control group (*p* < 0.01, Figure 1A). To rule out appetite-related weight loss, food intake was dynamically monitored during the administration period. The results indicated no statistically significant difference in food intake between the experimental and control groups (Figure 1B,C). The reduction in body weight in the high-dose group was positively correlated with body fat ratio and liver weight (Figure 1A,D,E). Conversely, the body weight trends for the other two groups (3 mg/day, 10 mg/day) showed no significant difference compared to the control group.

### 3.2. The Effect of Leflunomide on the Polysaccharides and Triglycerides in the Mice Livers

**Figure 2:**
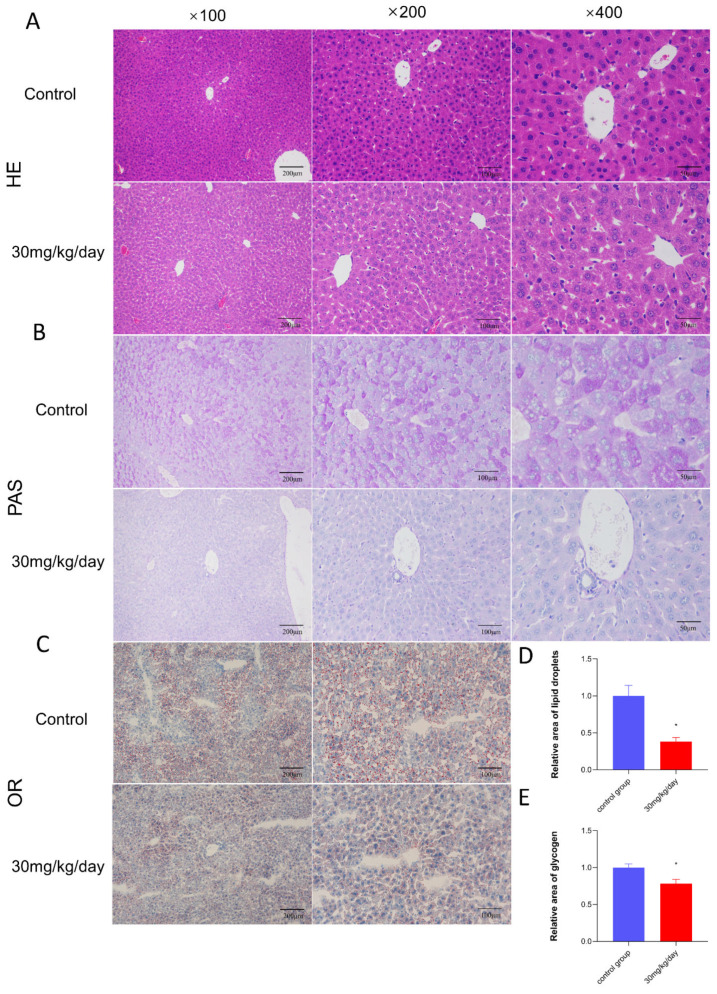
Morphological investigations of the liver of mice. (**A**–**C**) Scale bar of liver slices from mice in the 30 mg/kg/d group and control group. (**A**) HE staining of hepatic cells in each group. The control group showed normal morphological manifestation of hepatic cells. The arrangement and size of cells were normal. No steatosis or edema was found. The hepatic cells in the 30 mg/d group exhibited mild changes. The arrangement of cells was chaotic. Slight fat vacuoles could be observed in this group. (**B**) shows the quantitative data of PAS (periodic acid–Schiff) staining. (**C**) shows the quantitative data of oil red staining and triglyceride staining. (**D**,**E**) The staining results were quantified with Image J to show the levels of triglycerides and polysaccharides in each group. Leflunomide interferes with the synthesis of polysaccharides and triglycerides in the livers of mice. Data are expressed as mean ± SD, * *p* < 0.05.

Livers of the mice were collected for HE staining. The results showed mild liver damage in the 30 mg/kg/d group (Figure 2A). Fat amount is associated with glucose conversion in the body. Our findings demonstrated a reduction in body fat ratio in the 30 mg/kg/d group (Figure 2B). Triglyceride levels in the livers of mice administered 30 mg/kg/d for 12 consecutive days were significantly reduced compared to the control group (*p* < 0.05, Figure 2C,D). Fat synthesis is related to glucose levels in mice. Liver glycogen content was measured in the treated groups, revealing a significant reduction in liver glycogen content in the 30 mg/kg/d group after 12 days of continuous administration (*p* < 0.05, Figure 2B,D).

### 3.3. Leflunomide Inhibited the Biosynthesis of Aromatic Amino Acids

**Figure 3:**
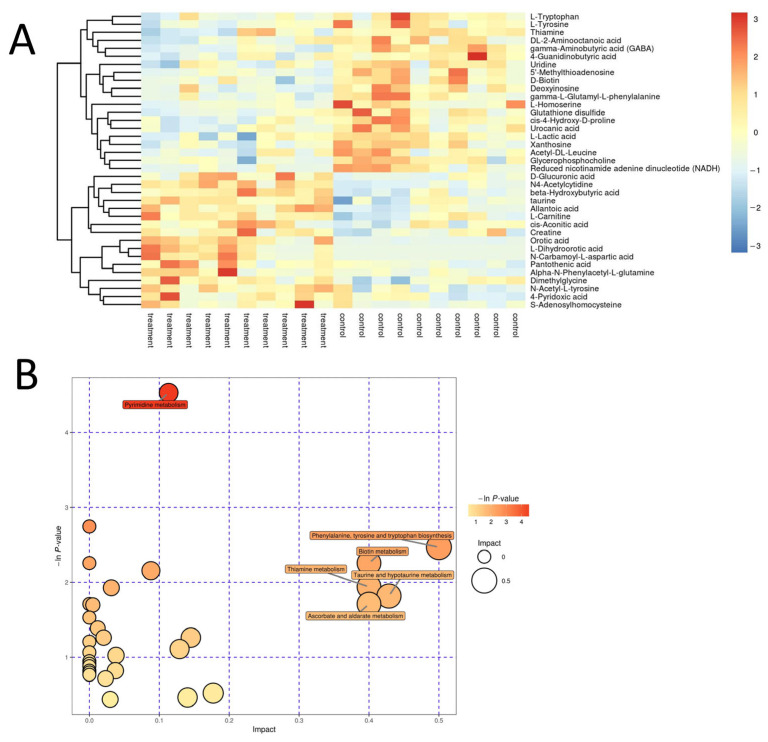
Leflunomide inhibits the biosynthetic pathway of aromatic amino acids in mice. (**A**) Heat map of the hierarchical clustering analysis of the treatment group to the control group. In the figure, the abscissa represents different experimental groupings, the ordinate represents the metabolic differences in this group, and the color patches at different positions represent the different expression levels of the metabolites at the corresponding positions. (**B**) Path analysis chart of treatment group to control group. The results of the metabolic pathway analysis are displayed in a bubble chart. Each bubble in the bubble chart represents a metabolic pathway. The abscissa and the size of the bubble indicate the size of the influence factor of the pathway in the topological analysis. The larger the bubble, the greater the influence factor. The vertical axis of the bubble and the color of the bubble indicate the *p* value of enrichment analysis (negative natural logarithm, namely, In *p* value).

Metabolomic analysis of metabolic phenotypic changes before and after administration revealed drug-induced changes in endogenous metabolites and associated them with adverse drug reactions. Liver tissues from the treated and control groups were analyzed, identifying 37 differentially expressed metabolites (Figure 3A). These metabolites were mapped to pathways in the KEGG database. Comprehensive analyses, including enrichment and topological analyses, identified metabolic pathways closely related to these differences (Figure 3B). Leflunomide primarily disrupted five metabolic pathways in mice: the biosynthesis of aromatic amino acids, biotin metabolism (vitamin H), thiamine metabolism (vitamin B1), taurine and hypotaurine metabolism, and ascorbate and aldarate metabolism (vitamin C).

### 3.4. Leflunomide Induces Endoplasmic Reticulum Stress in Hepatocytes and Causes Mice Liver Injury

**Figure 4:**
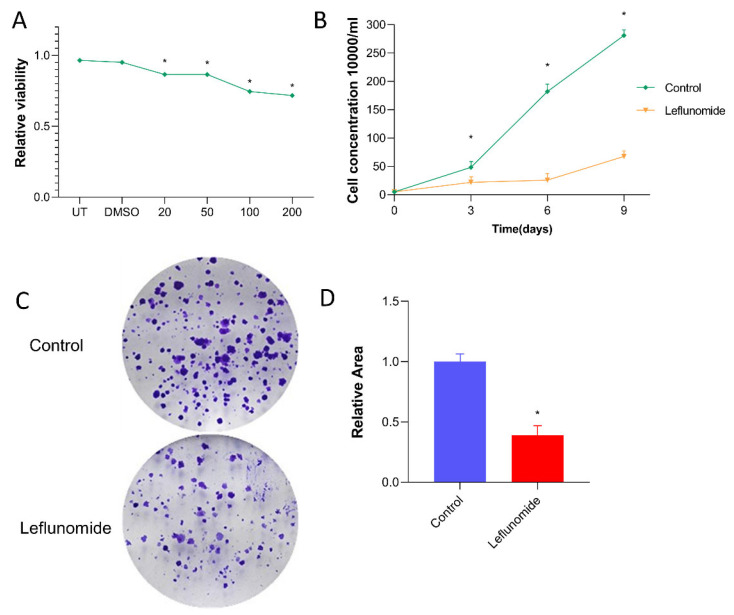
Leflunomide has proliferative toxicity to normal human hepatocytes. (**A**) Normal human hepatocytes were exposed to leflunomide at 20 µΜ, 50 µΜ, and 100 µΜ concentrations or DMSO, or were left untreated (untreated group: UT) for 12 h. Data are expressed as mean ± SD (n = 3). The concentration of 50% activity against liver cells (IC50 = 203 ± 6.78μM) was obtained from the metered response curve of leflunomide to normal human liver cells. (**B**) Normal human hepatocytes at a concentration of 10,000/mL were exposed to leflunomide at 20 µΜ for 9 days, and the fluid was changed every 3 days. (**C**) A total of 1000 normal human hepatocytes were exposed to leflunomide at a concentration of 20 µΜ, clone formation was observed after 9 days, and the medium supplemented with the drug was changed every 3 days. (**D**) Compared with the control group, the value-added activity of hepatocytes was significantly reduced in the leflunomide group, and this result has statistical significance. Data are expressed as mean ± SD, * *p* < 0.05.

Previous immunohistochemistry experiments revealed liver damage in treated mice. To investigate the cause of leflunomide-induced liver damage, similar in vitro cell experiments were conducted. Initially, leflunomide was applied to cultured human immortalized liver cells at gradient concentrations of 20 µM, 50 µM, 100 µM, and 200 µM. Leflunomide exhibited cytotoxicity at 20 µM (Figure 4A). Subsequently, cell function tests were performed on human liver cells at a concentration of 20 µM. The results indicated that leflunomide at 20 µM inhibited the proliferation of human immortalized liver cells (Figure 4B,C).

**Figure 5:**
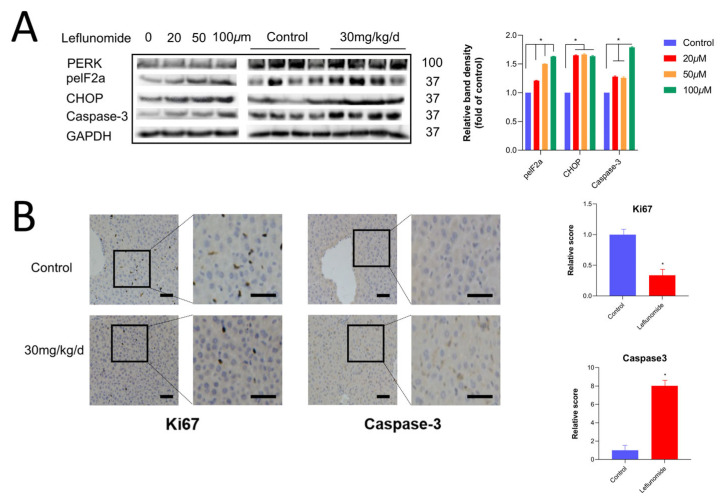
Leflunomide can induce endoplasmic reticulum stress. (**A**) After normal human hepatocytes were exposed to leflunomide at 20 µΜ, 50 µΜ, and 100 µΜ concentrations for 12 h. Immunoblotting experiments showed protein expression levels in cells or tissues and quantified the protein. On the right, there are four groups of mice in the control group and 30 mg/kg/d group randomly selected after 12 days to detect the protein levels in human normal hepatocytes in vitro of the liver. On the left side of the figure, there is the concentration of 20 µM. (**B**) Liver slices were immunostained with Ki67 and caspase-3 after 12 days in the control group and the 30 mg/kg/d group. The staining was then scored and quantified. Scale bars: left, 50 µm; right, 15 µm. Data are expressed as mean ± SD, * *p* < 0.05.

Following the inhibition of proliferation activity in human immortalized liver cells by leflunomide, the expression of endoplasmic reticulum stress-related proteins was investigated. These included protein kinase RNA-like endoplasmic reticulum kinase (PERK) and its downstream proteins phosphorylated eukaryotic initiation factor 2 (peIF2α), CCAAT/enhancer-binding protein homologous protein (CHOP), and the apoptosis-related protein caspase-3. The results showed that while the total protein level of PERK remained unchanged under leflunomide treatment, the expression of its downstream proteins CHOP and peIF2α increased. To establish consistency with the in vitro findings, in vivo experiments were also conducted (Figure 5A). Liver tissues of the experimental group mice were stained for the apoptosis protein caspase-3 and the proliferation protein Ki67. Compared to the control group, the 30 mg/kg/day leflunomide-treated group exhibited an increase in the apoptosis-related protein caspase-3 and a decrease in the proliferation-related protein Ki67, indicating liver damage in the treated mice (Figure 5B).

### 3.5. Leflunomide Inhibited the Activity of DAHPS in Intestinal Flora

**Figure 6:**
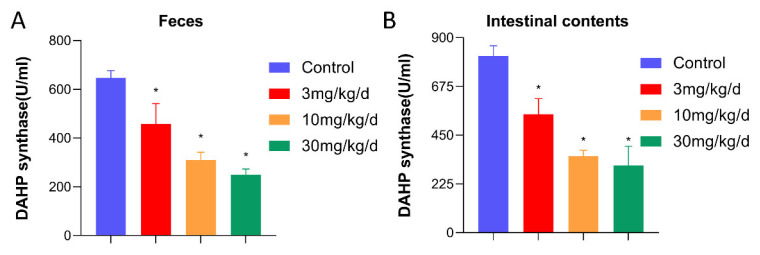
Leflunomide inhibits DAHPS activity in the intestinal flora of mice. (**A**) The feces of mice collected 12 days after the administration of the test were tested for DAHPS activity in the fecal intestinal flora. (**B**) Mice were dissected after 12 days of continuous administration, and the contents of the small intestines of mice were removed to detect DAHPS activity in the intestinal flora. Compared with the control group, these results have statistical significance. Data are expressed as mean ± SD, * *p* <0.05.

Metabolomic results show that leflunomide inhibits the synthesis of aromatic amino acids in the intestinal flora of mice. The biosynthesis of aromatic amino acids depends on DAHPS, one of the rate-limiting enzymes in this process [22]. Therefore, DAHPS activity was measured in the feces and gut microbiota of the experimental group mice. The results indicated that DAHPS activity in the feces and small intestine contents of the leflunomide-treated mice was significantly reduced (*p* < 0.05, Figure 6A,B)

## 4. Discussion

Leflunomide is widely used clinically, primarily for rheumatoid arthritis. Multiple reports have demonstrated that leflunomide can cause liver damage [23,24], and even hepatitis and liver failure [25,26]. This liver damage may be due to mitochondrial dysfunction. Leflunomide-induced mitochondrial dysfunction has been linked to endoplasmic reticulum stress [27]. Recent clinical data indicate that long-term use of leflunomide can cause adverse gastrointestinal reactions [23], typically attributed to the drug interfering with nutrient absorption in the gut. However, some studies suggest that these gastrointestinal reactions are not related to weight loss. The gut microbiota can metabolize nutrients that the human body cannot synthesize, providing essential amino acids and vitamins [13]. This study confirmed through in vivo and in vitro experiments that leflunomide inhibited the activity of DAHPS, a rate-limiting enzyme in the biosynthesis pathways of phenylalanine, tyrosine, and tryptophan, thereby inhibiting the biosynthesis of aromatic amino acids. This inhibition led to metabolic disorders and induced weight loss in mice.

In vivo studies demonstrated that weight loss in the experimental mice was closely related to the synthesis and interconversion of essential substances such as glucose, fat, and protein. The liver, being a key organ in metabolism, was significantly affected. Our results indicated that leflunomide interfered with the metabolism of triglycerides and glycogen in the liver. The liver weight of the treated mice also showed significant differences, suggesting that leflunomide might affect liver cell activity. Furthermore, there was no statistical difference in food intake between the treated and control groups. The observed daily food intake differences over four days might have been related to the mice’s stress response to external temperature.

In in vitro studies, leflunomide was found to inhibit the growth of normal human immortalized liver cells at concentrations of 20–50 μM. Measurement of the apoptosis-related protein caspase-3 showed increased expression, indicating that leflunomide promoted liver cell apoptosis. This mechanism may involve mitochondrial dysfunction caused by endoplasmic reticulum stress [27]. This study also reported that leflunomide triggered endoplasmic reticulum stress by stimulating the protein kinase R-like endoplasmic reticulum kinase (PERK) pathway, leading to increased expression of downstream proteins activating transcription factor 4 (ATF4) and CHOP, which resulted in mitochondrial dysfunction and apoptosis [28,29,30]. Irreversible cell apoptosis occurs if endoplasmic reticulum stress is not resolved [31], as confirmed by our findings. Under stress, the endoplasmic reticulum environment is disrupted, impairing protein maturation, and endoplasmic reticulum stress can regulate autophagy [32]. Autophagy, triggered by different types of cellular stress such as nutrient and energy deficiency, reactive oxygen species, and hypoxia, plays a crucial role in maintaining cellular homeostasis. Leflunomide induced weight loss by reducing fat deposition and inducing autophagy [33]. Our study demonstrated that leflunomide activated the c-Jun N-terminal kinase (JNK) pathway, although other reports indicated that leflunomide inhibited JNK activation [34]. Choudhury and colleagues [35] discovered that glutaminase expression was regulated through the JNK pathway, as demonstrated by chemical and genetic inhibition, and inhibition of glutamine decomposition can restore the mitochondrial function of senescent stem cells. The activation or inhibition of the JNK pathway may depend on the concentration of leflunomide and may be related to metabolism. The mechanism underlying these differential results requires further investigation

Leflunomide primarily interfered with the biosynthesis of aromatic amino acids. Among these, the pyrimidine metabolism pathway exhibited the most significant differences, which were related to the therapeutic effects of leflunomide. It was found that most of the differential metabolites in these pathways were essential substances that the human body cannot synthesize. In the analysis of differential metabolic pathways, the aromatic amino acid metabolism pathway showed significant differences in both topological and enrichment analyses. Phenylalanine, tyrosine, and tryptophan are three essential aromatic amino acids for the human body. Tyrosine can be derived from phenylalanine via hydroxylation and is the first degradation product of phenylalanine. Tyrosine metabolism produces fumarate and acetoacetate; fumarate can further participate in the tricarboxylic acid (TCA) cycle and gluconeogenesis. Acetoacetate can be involved in the synthesis of acetyl-CoA, influencing phospholipid synthesis and oxidative energy production. Tryptophan can be converted to acetyl-CoA through multiple steps and is a precursor of nicotinamide adenine dinucleotide (NAD). In our differential metabolite tests, NADH levels were significantly lower than in the control group. NADH is the reduced form of NAD, while NAD+ is its oxidized form. Tryptophan, nicotinic acid (NA), nicotinamide (NAM), nicotinamide mononucleotide (NMN), nicotinic acid riboside (NaR), and nicotinamide riboside (NR) are precursors of extracellular NAD+. Tryptophan can synthesize NAD+ through multiple steps [36]. NAD is crucial in the metabolic pathways that produce ATP, with NADH participating in oxidative phosphorylation and being directly involved in the transfer of hydrogen ions in this pathway [37], providing reducing equivalents for oxidative phosphorylation. A reduction in NAD directly impacts ATP production in cells. Our mouse model showed weight loss after administration. Metabolomic analysis of the mouse liver revealed that leflunomide disrupted the metabolic pathways of amino acids and vitamins. This result indicated that leflunomide interfered with the biosynthetic pathways of aromatic amino acids in the mouse liver. Further experiments demonstrated that leflunomide disrupted ATP production by inhibiting the activity of DAHPS in the mouse gut microbiota, thereby affecting the absorption and metabolism of the three amino acids.

In conclusion, our study revealed that leflunomide inhibited the activity of DAHPS in the gut microbiota, disrupting the metabolism of phenylalanine, tyrosine, and tryptophan, as well as the metabolism of carbohydrates and lipids. Leflunomide also increased endoplasmic reticulum stress by activating the PERK pathway, thereby promoting CHOP expression and increasing apoptosis-induced liver damage. These effects may be related to the observed weight loss induced by leflunomide.

## Data Availability

Data are contained within the article.
